# A Transdisciplinary Approach to Student Learning and Development in University Settings

**DOI:** 10.3389/fpsyg.2020.576250

**Published:** 2020-10-15

**Authors:** Nancy Budwig, Achu Johnson Alexander

**Affiliations:** ^1^Frances L. Hiatt School of Psychology, Clark University, Worcester, MA, United States; ^2^Department of Psychology, Anna Maria College, Paxton, MA, United States

**Keywords:** transdisciplinary, learning, higher education, development, interdisciplinary

## Abstract

This article considers the opportunities and challenges of transdisciplinary research on student learning in university settings. Fifty years ago, at a meeting in France that convened experts in education and psychology as well as higher education leaders, the term transdisciplinarity was coined as issues pertaining to the structure of the university and its impact on teaching and learning were considered. We argue that to move beyond what has already been discussed requires added insights from both the learning sciences and developmental sciences. In this article, these two areas are combined with the perspectives of higher education leaders. First, research is considered from the learning sciences on deep learning in relation to university learning and teaching. This body of work illustrates ways students need to be actively engaged in their learning and simultaneously frames teachers as facilitators of students’ constructive efforts rather than disseminators of static knowledge. Second, perspectives from the developmental sciences on processes of development are reviewed, focusing on adolescence and emerging adulthood. Here we highlight the importance of considering developmental systems approaches to aspects of organizing learning at universities in light of extensive research on adolescents and emerging adults. Third, we examine new higher education frameworks that have focused on the importance of student engagement, integration and application of knowledge and the implications of these shifts for organizing higher education learning in more holistic ways, often at the national and transnational levels. In reviewing these three areas, we consider what assumptions are made about the learner, the role of teachers and others in enhancing student learning, and the interaction between learners and contexts where learning takes place. We argue that while progress is being made in undergraduate reform efforts, implementation has been uneven. To deliver on this important work will require further alignment of the sort Jantsch (1972) and Piaget (1972) claimed was central to transdisciplinary approaches, namely aligning these different areas through a systems approach that considers education as a purposeful human activity. This will involve alignment and support from the learning and developmental sciences, as well as local, national and transnational efforts and learning communities to support campus efforts.

## Introduction

Fifty years ago at a meeting in France, Jean Piaget, and other scholars studying human development and knowledge, as well as higher education leaders gathered to speak about the importance of moving beyond the disciplines in considering university teaching and innovation. In fact, the term transdisciplinarity was coined and distinguished from multidisciplinarity and interdisciplinarity at that meeting (Apostel, 1972)^1^. Fifty years later, scholars and practitioners still are discussing the importance of a transdisciplinary approach to teaching and learning. Many of the challenges discussed at the original conference on teaching and learning ring as true today, and the question can be raised how to move forward to build on the original thinking, using the vast amount of research accumulated since that time in the learning and developmental sciences to guide this work. We will argue that progress can be made if these separate treatments of teaching and learning at universities are considered in a unified way.

Interdisciplinary and transdisciplinary approaches to knowledge have developed considerably over the last 50 years. While claims for similar problems identified at that meeting still exist (Bok, 2013), there is an increasing trend for openness to discuss new views of student learning, who our students are, and the goals of university education as they relate to societal needs and students’ professional and civic lives (Davidson, 2017; Wieman, 2017; Klemenèiè, 2019; Kamp, 2020). Furthermore, national and transnational involvement at the level of considering quality frameworks at the general level as well as within the disciplines help to mitigate some of the challenges of university silos and disciplinary limitations. We believe that simultaneous analysis of key foci of learning science and developmental science approaches when explicitly considered and aligned with current frameworks for innovation and advanced knowledge as they relate to organizing university structures and curricula is needed. Weaving together the sort of transdisciplinary approach Piaget and others imagined 50 years ago, holds promise to augment student learning and development, but also highlight the value of higher education in new and important ways.

## Three Perspectives on Student Learning and Development in Higher Education

### Learning Sciences: The Importance of Deep Learning in University Settings

The organization of teaching and learning in higher education has often been described as students passively absorbing material presented by an expert, drawing on processes of memorization, learning material in ways unrelated to what they already know, and often as disconnected from other learning within and between courses. We know from discoveries by learning scientists that these traditional views of learning and the pedagogies supporting them do not work in educational settings, and yet the vast majority of students experience this passive method of delivery in university classrooms. Furthermore, the 21st century needs citizenry and workforce able not only to master knowledge, but also create knowledge. For the last two decades, the Organization for Economic Co-operation and Development (OECD) and other organizations have stressed the importance of restructuring educational institutions based on theory and research from the learning sciences (Bransford et al., 2000). Graesser et al. (2008) produced an early and particularly rich list of 25 principles in an attempt to scale current learning research into various settings – whether K-12 schools, colleges, and lifelong learning. These principles suggest the importance of having students ask deep questions, highlight the assistance students need in self-regulating their learning, and advocate for anchoring learning in real world contexts important to the student. We also know from this body of work that students bring to their learning, not only a sense of agency but also their current understandings of topical areas. Learning is gradual and involves students revising their own intuitive understandings and change conceptual frameworks in light of new knowledge (Vosniadou, 2013, 2019).

In this section, we will examine learning science research with an eye toward learning in college classrooms with a particular focus on the cognitive underpinnings of learning^2^. To illustrate this point, we will focus on what learning scientists have called deep learning, looking into research on inquiry, the organization of knowledge, and metacognition to illustrate how learning scientists have focused on teaching and learning. Though we have accumulated a lot of evidence on how people learn, far too little of it has made its way into rethinking teaching at the college level (Budwig, 2013; Wieman, 2019). We will review some findings from this literature not to provide a thorough review (which is beyond the scope of this paper) but to consider how this body of work sheds light on the role of the student in learning, as well as the role of teachers in guiding learning.

#### Inquiry-Based Learning

Inquiry has been described as central to human learning in both formal and informal settings (Bransford et al., 2000). Student questioning actively engages the learner, as does students’ consideration of multiple solutions found in open-ended problem solving, which are both fundamental to student success (Darling-Hammond et al., 2019). When given the chance for exploration, students learn to frame interesting questions. While student-centered, inquiry has been noted to best be achieved when teachers provide guidance such as setting broad goals and encouraging students to focus on subgoals (Collins and Kapur, 2014). In fact, a meta-analysis of research on problem-based learning reveals many instances of students not learning when left completely on their own in formal learning settings (Alfieri et al., 2011). Thus, teachers play a critical role in selecting interesting problems of inquiry and providing high quality facilitation in order to produce learning outcomes (Walker and Leary, 2009; Lu et al., 2014).

The ability to inquire is something most college professors expect by the time students enter their classrooms and yet college students vastly differ in prior experience with practicing this capacity in formal learning settings. The tendency to approach formal learning contexts with an inclination to inquire often depends on the kind of schools students have attended prior to attending college (Kritt, 2018). Most college students have extensive practice with what Bloom (1956), has called “knowledge verbs,” that is, students have extensive practice with how to list, define, tell, and label information, but fall short in the capacity to inquire. These capacities are central to college learning, and yet many students arrive at and finish college insufficiently prepared to formulate appropriate questions and hypotheses, recognize assumptions and formulate premises, analyze, synthesis, and evaluate information, and formulate logical conclusions (see Eng, 2017). This lack of readiness has profound effects on students’ capacities for lifelong learning and professional engagement and has been noted in employer surveys in several recent studies (Archer and Davison, 2008, National Association of Colleges and Employers, 2017; Adecco, 2019).

#### Organizing and Generating Knowledge

Learning scientists have helped us understand learning and teaching by also contributing to an understanding of the importance of examining how students organize knowledge. Students must actively construct new knowledge building of their earlier novice conceptions (Piaget, 1978; Darling-Hammond et al., 2019). This implies that when teachers design learning environments, consideration must be made of what existing knowledge learners bring to the process of acquiring new information (Bransford et al., 2000; Sawyer, 2006). Novices (including most students) need significant help in developing the rich and meaningful knowledge structures central to high quality learning. In contrast to experts, novices have less complex and connected knowledge structures, making it difficult for them to process information in coherent chunks as experts do (Sawyer, 2006; Collins and Kapur, 2014). Learning science work has highlighted that in addition to the questions different disciplines engage in, each discipline has distinct ways of knowing. For example, it is important for students to not only know critical findings in science classes, but also that they deeply understand the ways scientists come to that knowledge, for instance, students need to grasp how scientists use models and representations. Following up on this, learning scientists have studied how students come to understand this. This body of work has highlighted the importance of focusing on learning principles guiding authentic experiences, including in the disciplines (Greeno and Engeström, 2014). The main point here is that the organization of knowledge is something students need to figure out, and research has suggested that in optimal teaching situations, the teacher scaffolds learning of both the content of new knowledge as well as practices engaged in by experts. This helps students increasingly and gradually acquire the capacity to engage in the practices of experts in the discipline.

#### Metacognition and Self-Regulated Learning (SRL)

The ability to inquire and organize knowledge is dependent on a third aspect of deep learning identified by learning scientists, namely metacognition and self-regulated learning. Metacognition put simply is thinking about one’s own thinking and involves a conscious attempt to regulate one’s own learning (Bransford et al., 2000). Examples of metacognition and self-regulated learning include thinking about ways individuals successfully learn, the necessary sequence of learning something, what one knows already and more importantly, what one does not know. Self-explanation and having the opportunity to explain your learning both to yourself and others has been noted to aid learning (Chi et al., 1994). It also has been helpful for learners to employ metacognitive strategies involved in reflecting on what one has learned, how what is learned relates to other knowledge, and ways to apply what is learned in different contexts. To this extent, metacognition can occur before, during and after a learning event and has been noted to enhance deeper understanding of the content learned (Zimmerman, 2000; Winne and Azevedo, 2014; Darling-Hammond et al., 2019).

A central question has been whether metacognition comes naturally or must be taught to learners. Evidence of metacognition has been found in preschoolers (Wertsch et al., 1980; Flavell et al., 1993) long before they enter formal schooling, when interacting on complex problems in the context of everyday interactions with others, though much research has highlighted that the breadth and depth of metacognitive awareness is something that develops well through adolescence. It has been noted that even many college students struggle with reflective practices involved with metacognition (Schraw and Moshman, 1995). It would seem as students begin college, opportunities to engage in metacognition would be extremely useful, since students are given much more autonomy for guiding their own learning on our campuses.

It has been shown that metacognition is learned in context as one engages in authentic problems (Palincsar and Brown, 1984; Bransford et al., 2000). Across age ranges, what holds constant is that metacognitive learning typically involves scaffolding or guidance with more experienced others (often experts) modeling or guiding how one draws on metacognitive strategies in the context of solving authentic problems in context. Central to the process of transferring agency for learning from expert to learner has been the use of specific symbolic tools which themselves come to scaffold the procedural steps guiding the learner to actively pull relevant information from complex settings through a series of prompts. Sometimes these tools involve the use of multimedia (Mayer, 2014) and other times, guidance is provided more directly through tools that provide classrooms with powerful mechanisms to guide reflection, often matching the kind of disciplinary practices engaged in by experts (Bielaczyc et al., 2013). These tools scaffold interactions and support learners by suggesting steps for practice and reflection as groups work together on improving one another’s ideas in classroom settings. Such tools have been used in elementary or secondary school classrooms in ways that help shift the classroom culture from a typical 20th century focus on dissemination of knowledge, to more active models of learning. The tools, employed in teacher-student dialogues, peer dialogues, as well as by learners themselves would seem to be useful in college contexts by helping to scale reflective practice in different disciplines by encouraging learners to engage in authentic inquiry, as well as integration and application of knowledge they are learning.

#### The Relation Between Students, Teachers, and Context in Learning Science Views of Deep Learning

While learning science research varies on a number of points, the views of deep learning described above share a similar perspective on the relation between learners and teachers characterizing their relationship as intricately linked and mutually influential. That is, deep learning involves an agentive learner who can actively draw upon their environments to examine, synthesize and build new knowledge. At the same time, research on the science of learning reviewed here has emphasized ways in which teachers and other experts, as well as mediational means and tools they employ support student learning. To this extent, learning scientists are both student centered and focused on the specific ways learning environments support student learning. More specifically, across all three areas (inquiry, organization of knowledge, and metacognition) while students actively engage in learning, it is a core aspect of learning science research to consider the specific and carefully sequenced ways teachers guide learning and gradually transfer increasing responsibility over to their students.

Learning scientists who have studied teacher knowledge (Shulman, 1986; Fishman et al., 2014) highlight the detrimental impact on learning when teachers have superficial pedagogical knowledge or content knowledge. Teachers may lack expert knowledge of the discipline or lack a solid understanding of the science of learning. To this extent, there is an important difference between instructors formally trained in the science of learning and formal experts who find themselves helping novices learn. For instance, instructors must consider whether students have appropriate prior knowledge and if so, whether and how students can activate that prior knowledge in order to learn new material. As experts, many college faculty underestimate this need (if they consider it at all) and thus do not spend time explicitly considering strategies to help students engage their prior learnings or revise inaccurate knowledge. Prior study of higher education teachers has revealed the positive effect pedagogical training can have on teachers (Postareff et al., 2007), as well as examples of how to improve classroom practices in light of learning science research (Ambrose et al., 2010). Challenges and barriers to faculty pedagogic training in this area has also been reported and is important to consider (Mälkki and Lindblom-Ylänne, 2012; Brownell and Tanner, 2017).

In sum, learning scientists have highlighted the intricate relation between student agency and how students draw on support from their environments. Especially for disciplinary learning in formal settings, students’ knowledge is built up gradually based on an assumption of constructivist effort on the part of the learner, and with simultaneous guidance by a knowledgeable expert who catalyzes student learning through carefully designing environments suitable for learning. Important for scaling efforts, learning science scholarship has also highlighted tools, such as guiding questions and protocols, can assist learners with more minimal intervention on the part of individual instructors, particularly relevant in larger classroom designs.

### Developmental Science Perspectives: Processes and Stages of Development Matter to Student Learning in University Settings

Developmental scientists examine behavioral and psychological aspects of human development. Recently there has been growing agreement that human development is best viewed from a systems perspective, as a process, with the organism viewed as inherently active (Witherington and Boom, 2019). In this section, we examine core features of developmental systems approaches, and then consider their application to stages of development relevant to college-attending students^3^. We use this developmental framework to examine identity formation and self-authorship during the adolescent and emerging adulthood years. Similar to our argument presented above, we will argue that college instructors rarely get any formal training about human development, and yet as we will argue, such knowledge is imperative to helping students learn. At the conclusion of this section, we will consider how developmental scientists view the role of the individual and environment in the complex process of human development and more specifically the relationship between student and teachers in our consideration of teaching and learning from a developmental lens.

#### Features of Developmental Systems Approaches

Systems theories provide a framework for understanding human functioning and development. The central claim relevant here is that development consists of multiple, interrelated processes that both affect and contribute to the dynamic organization of human systems (von Bertalanffy, 1972; Raeff, 2016). Rather than focusing on developmental outcomes or things that humans can and cannot do at particular ages, developmental systems approaches emphasize *developmental processes* involved in human functioning. Within a system, the developmental processes function as a whole (Raeff, 2016; Witherington and Boom, 2019). This appreciation of human organisms as functioning wholes, also presupposes constructivist accounts assuming individuals “are active agents in their own learning and development” (Amsel and Smetana, 2011, p. 4). To this extent, development does not stem directly from biological or environmental factors; rather individual and context are viewed as mutually influencing one another, as organisms actively engage in meaning construction (Overton, 2015; Lerner, 2016; Witherington and Lickliter, 2016).

#### Adolescence and Emerging Adulthood: A Holistic Examination of the Learner

While it seems common sense to assume that different stages of the life cycle are made up of distinct characteristics and abilities, theory and research stemming from developmental systems approaches have cautioned about developmental stage theories and milestones. What is central when looking at particular stages is the importance of *processes of development* and not simply outcomes, and to recognize that the developmental phases differ due to the ways individual, socio-historical, and cultural systems interact over time. With these caveats in mind, we turn to consider age-related developmental theories that are relevant to learning and teaching in university settings.

Those studying adolescence from a developmental systems approach, argue that what is distinctive during adolescence, is the nature of “adolescent *coordinating activities*” (Amsel and Smetana, 2011, p. 7, italics in original). Central here are ways in which organisms cognitively repackage what was present in prior organizational states. Many students who are still adolescents find the expectations of critical thinking and evaluation of contrasting points of view expected in college learning to be difficult. Instead, they readily accept ideas passed on by experts without critically evaluating them (Hodge et al., 2009). Furthermore, over the course of college students’ understanding of disciplines such as psychology and physics becomes more scientific with each year of majoring in that discipline (Amsel, 2018). Through ongoing attempts to make sense of their world, adolescents, as active agents, have opportunities, but also vulnerabilities if these co-ordinations are unsuccessful (Amsel and Smetana, 2011). For example, these vulnerabilities may show up with regard to academic underachievement (Crosnoe, 2011; Kuhn and Holman, 2011), as well as other areas such as vulnerabilities related to well-being, risk taking, and the like.

Following adolescence, Arnett (2000) argues for a distinctive stage in the lifespan that broadly represents the experiences of 18-29-year-olds (narrowly representing 18–25 year olds) as they transition into adulthood. Known as “emerging adulthood,” this stage is said to result from several demographic changes, one of which he notes is the increasing rise in the number of individuals of this age attending college. Arnett argues that of five features demarcating emerging adulthood, identity explorations is one of the most central as emerging adults explore a variety of areas including education, work, and love. The central point here is that developmental scientists not only examine ongoing processes of development, but also have identified core milestones and areas of interest that are in the foreground at particular junctures in the life cycle that influence and guide learning and development.

While adolescence has been noted to be a time of enhanced cognitive achievement for students, Arum and Roksa’s (2011, 2014) analysis of college-attending students suggests that college seniors spend only a minor amount of their time engaged in academics compared to time they spend socializing. Furthermore, they claim to have found only modest gains in critical thinking in emerging adults while in college. From a developmental lens, the question can be raised as to whether cognitive development has occurred in college and whether findings from the CLA test, which views cognition in isolation from every day and social settings in which it is embedded, is an appropriate way to test cognitive advances in this age group. Developmental scientists have focused on cognitive advances as part of larger processes and a developing system that includes areas like identity formation and self-authorship. The idea that during both high school and college students are pre-occupied with social relationships and questions of identity is hardly surprising to developmental scientists familiar with Erikson’s theory of development or Arnett’s (2000) portrayal of emerging adulthood. According to Erikson (1963) psychosocial stage theory of development, the fifth stage, which occurs during adolescence, is a time when teenagers explore questions of who they are and explore different roles and activities as they work to construct a sense of self.

#### Identity Formation and Self-Authorship in College-Attending Emerging Adults

A central claim we make here is that being in college helps emerging adults to engage in a period of identity exploration. It is an incubating period to try out multiple courses, majors, jobs, friends, and romantic partners before making more enduring choices (Arnett, 2016). College ideally provides a venue for individuals to explore and make long-term commitments in career, relationships, and worldviews (Arnett, 2016; Baxter Magolda and Taylor, 2016). Most importantly, college offers a fertile ground for exploring and developing skills and capacities that are necessary for making adult choices and decisions, central to this being the search for self. College attending emerging adults simultaneously engage with learning in new ways, dialoging with multiple others whose perspectives enlarge their worldview and offers the opportunity to practice skills that sets them on a path for lifelong learning as adults (Tanner et al., 2009).

A core aspect of identity exploration involves the search for a sense of self (Schwartz et al., 2016). College, if structured appropriately, can provide a space for students to engage their increasing cognitive capacities for abstract thinking toward this search as reflected in their consideration of multiple possibilities of who they are and what professions they can join in the future. Three such explorations where college provides a platform include taking on increased autonomy, developing cognitive acumen (e.g., critical inquiry, integration, and reflection), and finding identity-based work. Inherent in the aforementioned explorations of college-going emerging adults – autonomy taking, cognitive acumen, identity-based work – is the struggle to make meaning and write the initial drafts of their life stories (McAdams, 2013, 2016; Baxter Magolda and Taylor, 2016). These young people face questions of “Who am I,” “How do I relate to others,” and “What do I want myself to be” as they search for meaning in life and consider different worldviews, often through discussions with peers, faculty, advisors, and other staff who develop relations with students. Classroom interactions around intellectual and ethical issues offer students the opportunity to learn and select from multiple possibilities, which in turn broadens capacities for constructing a coherent life story (life authorship). These classroom and other academic experiences also can assist in developing internalized meaning structures (self-authorship). Both of these – life and self-authorship – are considered as important tasks during emerging adulthood (McAdams, 2013, 2016; Baxter Magolda and Taylor, 2016; Schwartz et al., 2016), with self-authorship central to autonomy taking (Baxter Magolda, 2001).

Self-authorship theory draws upon a constructivist framework by suggesting that young adults construct meaning in and through their interactions with the world. At this stage of development, emerging adults are passionate about compelling social issues such as social justice and equity. Self-authorship as a developmental process offers emerging adults the opportunity to connect the interactions between individuals, contexts and environments. On the pathway to self-authorship, individuals begin by blindly following and accepting formulas and knowledge presented by others, especially instructors. Over time, they come to realize the need to develop more autonomous values and beliefs in order to begin to robustly “author” one’s life. This involves arriving at a “comprehensive system of belief” (Baxter Magolda, 2001, p. 155) to guide life decisions (Baxter Magolda, 2001, p. 155). Baxter Magolda argues that learning environments are central to self-authorship especially when teachers give agency to students and downplay their own authority, situate learning as a process that is relevant to students’ experiences, and provide examples of teachers’ modeling thinking and learning processes, while also encouraging significant space for student reflection (Baxter Magolda, 2008).

#### The Role of Students, Teachers, and Context in Dynamic Theories of Development

Because scholars of human development have emphasized the importance of examining human systems, rather than isolated developments (cognitive, social) of human functioning, student development must be looked at holistically and not simply in terms of the cognitive structures and processes that students bring to the classroom. Students enter the classroom not just with a mind, but fully embodied to engage with their surroundings, including seeing the classroom as a social activity. For instance, adolescents have significant challenges in coordinating budding knowledge systems and understanding the difference between facts and theories. In addition, adolescent and emerging adult students’ preoccupation with social relations, identity and work are all factors relevant to understanding student learning as it is being considered in university settings. In particular, this age group is particularly interested in weaving their academics with issues of social concern, identity, and work.

Teachers also need to take into account that their students are continuing to develop such that the cognitive abilities of first year students differ significantly from seniors often in the same class. Furthermore, college access has changed such that there exists tremendous variation in individual differences in learning in a given class as well (Gagné, 2005). Universities have long overlooked this, grouping students together without considering these differences that can be productively used to augment teaching and learning. Those entering college are continuing to coordinate prior systems of development in new ways, use their burgeoning ability to reflect in increasingly abstract ways, and all of this is centrally linked to their exploration of identity, which is far more developed by senior year of college. While developmental scientists acknowledge the role of others in students’ development, compared to the other perspectives in this article, their work focuses more on the individual’s own construction of knowledge and efforts to engage in meaning making.

### Higher Education Perspectives: Reimagining the Undergraduate Degree and Learning Outcomes Within the Disciplines

Higher education leadership is a third group that has played a significant role in considering the learning experience of students at colleges and universities. After a flurry of activity in the 1960s and 1970s (Apostel, 1972; Levine, 1980), new issues emerged as universities have been noted to serve a much broader set of regional and governmental needs (Davies et al., 2001), and with a broader range of students attending college, many underprepared for the curriculum offered (Baum and McPherson, 2019). Neoliberalism and models of higher education that are said to treat universities more like corporate organizations have become more the norm (Taylor, 2017). Such trends not only encourage specialization and compartmentalization, but have posed challenges to developing a view of higher education in terms of lifelong learning, student agency, and education has shifted from a public good to a private good.

Fresh discussions about curricular models have been increasing during the first decades of the 21st century, with some at the level of institutional planning, while global initiatives have brought together individuals from around the globe (Elkana et al., 2010; Elkana and Klopper, 2016). The most enduring reform efforts have been tied to multi-institution and governmental platforms, with the most ambitious scalability effort in the first decade of the 21st century known as the Bologna Process. The primary goal of the Bologna Process has been to bring more cooperation between countries within the European Union with the aim to increase mobility and increase recognition for a coordinated European higher education system (Zahavi and Friedman, 2019). In the United States, national attempts to reimagine a vision for higher education built on the enduring aims of a liberal education but simultaneously connecting that vision more clearly to the complex challenges of our world (Schneider, 2008) also gained significant momentum. Each of these approaches had a different relationship to the disciplines where reimaging and reform also has taken place. We turn to consider these efforts and then provide a discussion of their mutual implications for considering the relation between students, teachers, and contexts in university settings.

#### Visions for Undergraduate Education: New Learning Outcomes for the 21st Century^4^

The Bologna process noted above has been said to be one of the most ambitious credible attempts to scale for accountability in higher education (Adelman, 2008). Working at three levels (transnational, national, and disciplinary), a central feature of this work has been the establishment of a quality framework. Prior to this, degrees were awarded without much attention to the quality of learning. By 2003, a set of core competencies were proposed. Known as the Dublin Descriptors these included: Knowledge and understanding; Applying knowledge and understanding; Making judgments; Communication; and Lifelong learning skills. Adopted in 2005 as the Qualifications Framework of the European Higher Education Area, work shifted to implementing the common set of learning outcomes as a mechanism of improving the quality of an undergraduate degree in a transparent and holistic way across European countries. Notable here within the European context, was the decision to focus on quality assurance through what has been called the Tuning project, where tuning takes place at the levels of individual disciplines. This work has not been without its challenges that also will be discuss below (Reichert and Tauch, 2005; Kehm, 2010).

During the same period Americans also have aimed to address quality issues. National associations have been active in this arena, most notably the Association of American Colleges and Universities (AAC&U) through its Liberal Education and America’s Promise (LEAP) initiative. Although for many liberal education has been associated with small residential colleges, the definition of the difference between liberal arts colleges and liberal education has been the subject of recent clarification. Liberal arts colleges are typically small and residential, while the modern notion of liberal education extends beyond particular features, or the kind of students who chose to attend those schools. Liberal learning has long been unified as an approach that promotes breadth and depth of knowledge, intellectual skills of inquiry and analysis, and personal and social responsibility. In the recent 15 years, a fourth learning outcome promoting the integration and application of knowledge has been added. Schneider (2018) argues that the aim is for all university students to experience liberal learning and not just students who either attend particular kinds of institutions, or who major in particular disciplines (such as the arts and humanities). According to Lynn Pasquerella, President of the Association of American Colleges and Universities (AAC&U, 2020, p. 2) “AAC&U remains steadfast in our conviction that a liberal education offers the best preparation for work, citizenship, and life.”

One striking example of the more integrated and applied academic experiences encouraged to be at the heart of a liberal education involves student participation in what Schneider refers to as a signature work project (Schneider, 2015). Such an experience involves an extended project (at least 6 weeks of work) that reflects “cumulative and integrative learning across general and specialized studies” (Schneider, 2015, p. 6). In addition, the project should connect to a significant problem that has no clear answer and require significant student agency to solve in a way that is meaningful to the student and society, often as part of a capstone experience (Peden, 2015; Schneider, 2015). Central to the aspiration of signature work is the idea that all college students, and not just the very best, would actively engage in an integrative and applied project before leaving college. The call for applied and project based work for all college students, one that activates the agency and imagination of students can be found transnationally (Elkana and Klopper, 2016; Kamp, 2020).

#### Reimagining the Disciplines

As noted above, The Tuning Project locates reform efforts within the disciplines, leaving disciplines to rethinking teaching and learning. Wieman (2019, p. 65) speaking about STEM fields argues: “The acquisition of basic information is now of limited value, while complex reasoning and decision-making skills that can be broadly applied have high value in many aspects of modern society.” More specifically, expertise in a discipline involves a set of cultural practices often not explicitly discussed. Until recently, teaching in the disciplines has been taken as a solitary activity left to individual tastes and styles. Current discussions in various disciplinary groups have begun to stress the importance of helping students think like an expert in the discipline, using the tools and complex reasoning that experts in a discipline employ (Wieman, 2019).

Historians have also become more explicit about the learning outcomes for the discipline. According to the American Historical Association’s Tuning Project (2016) learning history is more than dates, and rather involves “a deliberative stance toward the past; the sophisticated use of information, evidence, and argumentation; and the ability to identify and explain continuity and change over time. Its professional ethics and standards demand peer review, citation, and acceptance of the provisional nature of knowledge.” With direct traces to the Quality Framework Tuning Project discussed above, the American Historical Association has provided faculty with tools and resources to engage in forward moving conversations in order to reinvigorate the classrooms for students in learner-centered ways around an explicit set of disciplinary learning outcomes that are tied to enhancing student agency.

While other disciplinary groups have similarly adopted new learning outcomes, one particularly forward looking attempt is that provided for engineering education developed by Kamp (2020). Not only are quality frameworks with student learning outcomes outlined, but one finds explicit discussion of working between the gap of broad vision and on the ground implementation of educational reform. Kamp outlines both the mindsets and competencies needed by engineers in the 21st century, and highlights the important role that students must play in their own educational process, recognizing that students bring to their learning a very different approach than students of the past. According to Kamp (2020), navigating engineering education must be viewed as a lifelong process, with individuals knowing how to continuously relearn given new contexts and developments.

Combined work going on in the disciplines points to the importance of linking both disciplinary knowledge and expertise with core outcomes that go beyond any single disciplinary field. This body of work highlights enormous new responsibilities and roles for teachers as they transition to more learner-centered strategies, which makes this work rewarding but challenging.

#### The Structural Changes Necessary to Deliver on This New Vision of Student Learning in Higher Education

Those in the higher education literature have been highly attuned to the processes and structures necessary for implementing the new vision of student learning. identifying at least three difficulties. Already by 1970, members of the OECD conference on interdisciplinarity noted that the siloed nature of higher education, with its focus on disciplinary units would make more integrative and applied models of learning difficult to implement. Higher education institutional structures are set up around disciplinary knowledge and practice and these structures, as well as the extensive disciplinary training experienced by faculty in such departments constrains interdisciplinary transformations. The Tuning Project and some of the work going on through disciplinary societies linked to overarching learning outcomes in part has worked to address this challenge. A second issue considered by universities as they have attempted to incorporate a curricular framework supporting student-centered learning is that institutions vary tremendously in their mission and goals and as such each campus working on such an implementation will look different – one size does not fit all, again making institutional change difficult. As Davies et al. (2001) have noted, leadership matters and there has been some conflicting messages about the importance of equity and learning along side what has been called an “arms race” of elitism, especially tied to research excellence that are in tension (Kehm, 2010). A third challenge identified is that the student-centered learning and quality delivery of ambitious outcomes require extensive time and effort on the part of faculty. Looking at the implementation of quality standards as part of the Bologna Process, Reichert and Tauch (2005) have noted the importance of campuses finding their own ways in. Similarly, AAC&U has organized cohorts of schools under grant funded initiatives such as *Faculty Leadership for Integrative Liberal Learning* and the *LEAP Challenge: Building Capstone and Signature Work*, facilitating and supporting institutions as they created and scaled the kind of integrative and applied learning experiences. In addition, a set of resources has been created through reporting out regularly on findings for other schools to draw upon (Ferren and Paris, 2015; Budwig and Jessen-Marshall, 2018).

#### The Relation Between Students, Teachers, and Contexts in University Settings

Higher education assumes the importance of students’ constructive efforts and the importance of faculty leadership for designing environments where learning can flourish. Higher education also has given significant attention to the role of university systems and processes to support the teaching and learning efforts at universities. A growing trend in higher education is to emphasize the importance of shifting from what teachers do (e.g., teaching) to what students learn (Wright, 2011). While endorsing this view, Wright (2011) argues that it is important to recognize that leaving students with more responsibility and agency for their own learning is not an easy pivot for higher education to make (Wright, 2011). While there is recognition of the importance of student-centered learning and student agency in the construction of knowledge, the bulk of the discussion by higher education leaders has focused on the guidance received not only by individual faculty and staff, but also through intentional institutional design.

Higher education discussions primarily have focused on the need for organizational supports and structures to aid in assuring the necessary dynamic between student agency and engagement and faculty support and guidance. University leaders have recognized the lack of training faculty bring with them regarding teaching and learning in general, and for integrative and applied learning in particular. As universities have begun to strategically emphasize student-centered approaches to learning, teaching and learning centers have been built up on campuses as cross-disciplinary spaces to support and nurture quality teaching (Hutchings et al., 2011). At the same time, as one turns to more holistic approaches to student learning, and the importance of student agency and lifelong learning has led to consideration of the role others can play in student learning highlighting the need for consideration of more complex institutional structures and non-academic supports needed. Recognizing the limited feasibility of charging faculty with sole responsibility for student learning there is need for coordination when students are expected to integrate their learning and apply it to problems that often involve participation beyond university gates.

## Discussion: Opportunities of and Challenges for a Transdisciplinary Approach to Learning and Development in University Settings

As noted in the introduction to this article, 50 years ago higher education leaders and professors came together to discuss teaching and learning in higher education. At the original seminar, and subsequent publication from this important meeting, the terms multi-, inter-, and *trans-*disciplinarity were coined and debated (Apostel, 1972). Piaget argued for the need to situate the discussion in the context of epistemological views of knowledge. Building off of Piaget’s structural approach, Jantsch (1972) argued for a view of knowledge more strongly linked to practice: “A systems approach (that)… would consider education and its motivation, above all, as … a purposeful human activity” (p. 99). The seminar and subsequent publication tied the problems and necessary solutions to stronger examination of institutional structures and reorganizations to simulate further work in this area. While there is no doubt that issues of interdisciplinarity and transdisciplinarity have led to a significant amount of discussion, the question raised here is whether it has impacted work on transdisciplinary approaches to learning and development in university settings.

The research topic guiding the papers in this issue of *Frontiers* starts from the assumption that in the area of research on learning and teaching, there has been significant effort to engage in interdisciplinary and transdisciplinary research in the fields of education, psychology, and learning sciences. Nevertheless, it is argued that an obstacle has been in breaking out of those silos to integrate those findings. Building on this claim, this article has brought together three perspectives never before considered side by side. The findings of each area are not new, but a careful examination highlights important insights when linking distinct areas of thought. The lens from the learning and developmental sciences, when placed next to higher education practitioners bring unique vantage points to our understanding of student learning, the role of teaching, and their interaction within larger university systems of which they are a part.

The opportunity of piecing together the distinct areas is precisely what Piaget (1972) described, namely the attempt to understand the development of knowledge within a systems framework, with each part contributing a level of analysis. Piaget’s (1972) abstract view of knowledge did not take up on the situatedness of learning and the important role real world problem solving plays for college students. Theory and research from the learning and developmental sciences offer fresh perspective. Across all three areas (learning sciences, developmental sciences, and higher education), there were important areas of agreement in discussions of learning and teaching in university settings. All groups ideally take a constructivist approach to learning and development, sharing the belief that students must be viewed as agents of their learning and development. Furthermore, there is agreement across all three groups that more experienced others should focus less on teaching and disseminating set knowledge and practices to newcomers, and rather aim to be guides helping scaffold student engagement. Furthermore, all three groups recognize the importance of larger ecosystems in learning and teaching, with learning science research focusing on classroom design and disciplinary guidelines, developmental sciences examining student background, interaction with peers and others, and higher education focusing on disciplinary and university contexts and broader quality goals of modern higher education to assure citizenry and workforce readiness. We conclude by suggesting that if the views of students as learners, the importance of considering students’ civic and professional identity formation, and university and disciplinary learning outcomes by national, transnational and disciplinary groups are aligned this sort of systems approach could mitigate some of the challenges that have been discussed. Work to date has shown that systems of support at the disciplinary, campus, national, and transnational levels and especially learning communities assist in bringing about necessary undergraduate reforms.

While there was shared agreement across the different areas, each area studies learning and teaching with different amounts of focus. Furthermore, the joint consideration of all three areas provides added insights into future avenues of work. For instance, the learning science focus on deep learning in general (and inquiry, integration, and metacognition in particular), illustrates how delineating what it means to know impacts the design of learning environments in university settings to support student learning. Learning scientists also have revealed the guiding role of teachers and others in creating particular kinds of contexts where learning happens. From the work of developmental scientists, we have a much deeper appreciation of what individual learners bring with them to the classroom at distinct periods of the lifespan. Particularly salient for issues of learning and teaching in university settings is the understanding of the ways cognitive and social development interact with learning and teaching. For example, developmental science has highlighted adolescents’ and emerging adults’ growing ability to entertain the multiple perspectives often presupposed by university teachers, and their nascent abilities to self-author their developmental trajectories influencing and influenced by their interest in making society a better place. The higher education work has highlighted how important consideration of the university eco-system and its ties to national and transnational efforts is to progress in learning and teaching in university settings. This work highlights structural and other aspects of learning and teaching, such as the extensive focus on disciplines, the power of disciplines and autonomy of individual faculty to teach as they see fit, and the lack of faculty training in teaching and learning as examples. Ongoing learning communities are needed to cultivate and support faculty efforts.

**FIGURE 1:**
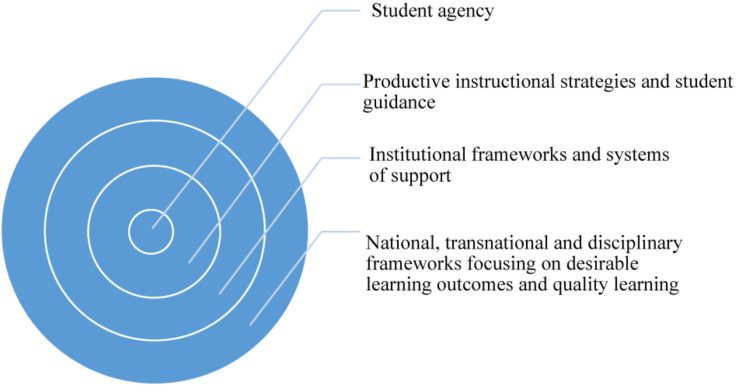
A transdisciplinary approach to student learning.

A systems approach, pulling together these disparate levels of analysis from the learning sciences, developmental sciences, and higher education, provides a powerful way forward and work against neoliberal fragmentation. Figure 1 depicts the nested relationship between these different dimensions and their nested connections.

This work though is not without its challenges, challenges that were articulated 50 years ago, and which serve the neoliberal university well. Nevertheless, these issues can be freshly addressed by the transdisciplinary areas described in this article. Table 1 provides an overview of the goals of reform efforts, main attributes, opportunities, and challenges of each of these fields.

**TABLE 1:** Fields, main attributes, opportunities, and challenges.

Field	Main attributes	Opportunities	Challenges
Learning sciences	Interaction between students as inquirers and productive instructional strategies for deep learning	Extended knowledge about how students learn Emphasis on lifelong learning as a way to approach the complex and changing world and learners’ role in problem-setting and problem-solving Centers for excellence in teaching and learning	Most faculty focus on “teaching” and lack explicit training in student learning and productive teaching practices Primary and secondary education has not set all students up equally for higher education learning
Developmental sciences	Recognition of the connection between students’ construction of knowledge and development of agency and identity formation Recognition of emerging adults searching for meaning and purpose in work and civic life	Extended knowledge about emerging adulthood Importance of holistic approach to development Recognition of the importance of viewing learning as a process and deeply connected to identity formation	Instructors lack explicit knowledge about student development Academic support services have such background knowledge on student development but universities often lack mechanisms to coordinate academic units with student facing support services at the university
Higher education	Leadership matters: disciplinary societies, national and transnational organizations, and individual universities set agenda, frameworks, and outcomes for student learning	University leaders and academic organizations explicitly address these matters rather than leaving them to individual faculty preferences and styles Alignment across disciplinary, university, national and transnational goals New thinking about cultures of learning and role of students, faculty and staff in that work	Learning as a public good is often in tension with “arms race” approach to university rankings, which often focuses on grants and research Reward structures Organizational design based on silos, and university procedures and policies are not well aligned with modern understanding of student learning and development

*Purpose: A transdisciplinary approach to learning aims to enhance students’ ability to be lifelong learners, creating systems of support to bring about curricular reform.*

The most major challenge is how to work with the training and reorganization of universities to allow integration across these levels of analysis to happen. At the undergraduate level, significant work is already underway to rethink curricular structures (e.g., the work reviewed above to create overarching learning outcomes associated with an undergraduate education). More directly linking this work to what is currently known from the learning and developmental sciences would provide fresh answers absent in the work identifying this problem 50 years ago. A transdisciplinary approach would also require significant changes to doctoral training, assuring that the next generation of faculty receive training in modern day learning and developmental science and are prepared for their roles as teachers. In addition, more work is needed to examine current university structures and rewards based on a transdisciplinary approach to learning and teaching to be sure our institutions are ready to support optimal learning and value excellence and success in student learning. Enhancement of opportunities for students and faculty to work collaboratively with other units on campus, and for universities to build partnerships beyond the campus gates have been highlighted as important as well. While these challenges are significant, the progress made in the last decades in the learning and developmental sciences show promise for new answers to questions that have been identified and stubbornly resistant to change. This issue of *Frontiers* symbolically represents one extremely important change necessary to move this dialogue forward.

It seems clear that higher education is moving closer to a vision of higher education that entails a common agenda- one that values broadening access, considers quality enhancements, and views higher education as a public good. More difficult has been figuring out how best to implement this common agenda and we have seen different approaches in the United States and within European countries. There are some commonalities suggesting best practices for sustained change. For instance, individual, institutional, and regional efforts have worked best when implementation involves active participation of individuals who not only adopt but adapt a broad agenda in contextually relevant ways. Consistent support rather than sanctions have aided implementation efforts, whether the sort of learning communities formed through the AAC&U learning institutes or collectives of institutions working on common problems, or the networks of support formed in both Europe and the United States as communities of learners similarly work together to construct resources and guidance. A close examination of these efforts shows that macro-level implementation efforts at the national or transnational level are working better than the microlevel change taking place on individual campuses (Sabatier, 2005; Pálvölgyi, 2017; Budwig and Jessen-Marshall, 2018). This highlights the importance of reviewing local efforts, and whether the support systems and guidance are in place to promote this work. Interestingly, this theory of higher education change involves precisely the sort of principles and frameworks advocated for student learning and that was suggested by Piaget and Janatsch, namely a systems approach that considers education and its motivation in transdisciplinary ways as a purposeful human activity, not only for students but for faculty, staff, and administrators guiding that change. The next logical step would entail a workshop like that 50 years ago with explicit discussion of the benefits of the learning and developmental sciences, alongside what we are learning from higher education reform efforts.

The work ahead is complex but can be justified in that it is exceedingly important at this juncture. Education holds the possibility to positively change society in transformative ways. As noted 50 years ago by Piaget (1972, p. 103): “If education is accepted as being essentially education for the self-renewal of society, it becomes an important, or even the most important agent of innovation.” Viewing education as a public good, and innovation as intricately tied to education makes building a transdisciplinarity exemplar around the topic of teaching and learning at the university level a particularly important area of scholarship to work on. To enact the levels of analysis identified in this paper and address the challenges, it will be necessary to not only consider contributions from the learning sciences, developmental inquiry, and efforts of higher education leadership side by side, but also how best to align them in ways that assure not only a compelling vision, but also successful implementation.

## Author Contributions

NB contributed the original conception of the study, wrote the sections of the manuscript, and focused on learning sciences and higher education. The authors worked collaboratively on the section on developmental science with AA playing a major role in writing the section on emerging adulthood and self-authorship. Both authors contributed to manuscript revision and approved the submitted version.

## Conflict of Interest

The authors declare that the research was conducted in the absence of any commercial or financial relationships that could be construed as a potential conflict of interest.

## Footnotes

1 Piaget (1972, p. 136–138) used “interdisciplinarity to designate… cooperation among various disciplines lead to actual interactions, to a certain reciprocity of exchanges resulting in mutual enrichment. “This was distinguished from the term he considered a higher stage- “transdisciplinarity”, … (would) place these relationships within a total system without any firm boundaries between disciplines.” These two terms contrast with multidisciplinary approaches which simply juxtapose different disciplinary contributions. Thirty years after the original conference where these terms were coined, a further conference took place and a new consensus emerged on how to define transdisciplinarity. Klein elaborated the systems approach of Piaget by including practitioners outside a given discipline. “The core idea of transdisciplinarity is different academic disciplines working jointly with practitioners to solve a real-world problem. It can be applied in a great variety of fields” (Klein et al., 2001: 4). This is the approach that we will adopt to transdisciplinarity in this article.

2 Elsewhere we have discussed social aspects of learning and its relation to college learning (see Budwig, 2013, 2015).

3 We focus here on traditional age college students, typically 18–25 year olds, recognizing that adult learners, while not the majority, are a growing group attending college.

4 While our focus here is on Europe and the United States, important work in this area is going on elsewhere [see for instance, Godwin (2015); Al-Hendawi and Albertine (2019)].
